# (+)-Limonene 1,2-Epoxide-Loaded SLNs: Evaluation of Drug Release, Antioxidant Activity, and Cytotoxicity in an HaCaT Cell Line

**DOI:** 10.3390/ijms21041449

**Published:** 2020-02-20

**Authors:** Eliana B. Souto, Aleksandra Zielinska, Selma B. Souto, Alessandra Durazzo, Massimo Lucarini, Antonello Santini, Amélia M. Silva, Atanas G. Atanasov, Conrado Marques, Luciana N. Andrade, Patricia Severino

**Affiliations:** 1 Faculty of Pharmacy (FFUC), Department of Pharmaceutical Technology, University of Coimbra, Pólo das Ciências da Saúde, Azinhaga de Santa Comba, 3000-548 Coimbra, Portugal; zielinska-aleksandra@wp.pl; 2 CEB—Centre of Biological Engineering, University of Minho, Campus de Gualtar, 4710-057 Braga, Portugal; 3 Department of Endocrinology, Hospital de São João, Alameda Prof. Hernâni Monteiro, 4200-319 Porto, Portugal; sbsouto.md@gmail.com; 4 CREA-Research Centre for Food and Nutrition, Via Ardeatina 546, 00178 Rome, Italy; alessandra.durazzo@crea.gov.it (A.D.); massimo.lucarini@crea.gov.it (M.L.); 5 Department of Pharmacy, University of Napoli Federico II, 80131 Napoli, Italy; asantini@unina.it; 6 Department of Biology and Environment (DeBA-ECVA), University of Trás-os-Montes e Alto Douro (UTAD), Quinta de Prados, 5001-801 Vila Real, Portugal; amsilva@utad.pt; 7 Centre for Research and Technology of Agro-Environmental and Biological Sciences (CITAB), University of Trás-os-Montes e Alto Douro (UTAD), 5001-801 Vila Real, Portugal; 8 Institute of Neurobiology, Bulgarian Academy of Sciences, 23 Acad. G. Bonchev str., 1113 Sofia, Bulgaria; atanas.atanasov@univie.ac.at; 9 Institute of Genetics and Animal Breeding, Polish Academy of Sciences, Jastrzębiec, 05-552 Magdalenka, Poland; 10 Department of Pharmacognosy, University of Vienna, Althanstraße 14, 1090 Vienna, Austria; 11 Ludwig Boltzmann Institute for Digital Health and Patient Safety, Medical University of Vienna, Spitalgasse 23, 1090 Vienna, Austria; 12 Laboratory of Nanotechnology and Nanomedicine (LNMED), Institute of Technology and Research (ITP), Av. Murilo Dantas, 300, 49010-390 Aracaju, Brazil; conrado.marques@souunit.com.br; 13 University of Tiradentes (UNIT), Industrial Biotechnology Program, Av. Murilo Dantas 300, Aracaju 49032-490, Brazil; 14 Tiradentes Institute, 150 Mt Vernon St, Dorchester, MA 02125, USA; 15 Laboratory of Nanotechnology and Nanomedicine, Institute of Technology and Research, Aracaju SE 49032-490, Brazil; luciana.nalone@hotmail.com; 16 School of Pharmacy, University Tiradentes, Aracaju SE 49032-490, Brazil

**Keywords:** (+)-limonene 1,2-epoxide, monoterpene, Imwitor 900K-SLN, lipid peroxidation, cytotoxicity, HaCaT cell line

## Abstract

In this work, we developed a solid lipid nanoparticle (SLN) formulation with (+)-limonene 1,2-epoxide and glycerol monostearate (*Lim*-SLNs), stabilized with Poloxamer^®^ 188 in aqueous dispersion to modify the release profile of the loaded monoterpene derivative. We also evaluated the role of SLNs in lipid peroxidation and cytotoxicity in a spontaneously transformed aneuploid immortal keratinocyte cell line from adult human skin (the HaCaT cell line). For the cell viability assay, the colorimetric 3-(4,5-dimethyl-2-thiazolyl)-2,5-diphenyl-2H-tetrazolium bromide (MTT) assay was used. *Lim*-SLNs with a loading capacity and encapsulation efficiency of 0.39% and 63%, respectively, were produced by high pressure homogenization. A mean particle size of 194 ± 3.4 nm and polydispersity index of 0.244 were recorded for the loaded *Lim*-SLNs, as compared to 203 ± 1.5 nm (PI 0.213) for the non-loaded (blank) SLNs. The loading of the monoterpene derivative into glycerol monostearate SLNs fitted into the zero-order kinetics, and ameliorated both lipid peroxidation and cytotoxicity in a keratinocyte cell line. A promising formulation for antioxidant and anti-tumoral activities is here proposed.

## 1. Introduction

Natural products obtained from the essential oils of medicinal plants are a recognized source of ingredients in traditional medicine [1]. Compounds of natural origin have indeed been the source of commonly used chemotherapeutic drugs, e.g., vinblastine (an alkaloid from *Catharantus roseus* [2]), camptothecin (an alkaloid extracted from the bark of *Camptotheca acuminate* [3]) and its derivatives (irinotecan and topotecan), etoposide (a semi-synthetic derivative of 4’-demethylepipodophyllotoxin, a naturally occurring compound produced by *Podophyllum* spp. [4]), and paclitaxel (a tetracyclic diterpenoid originally isolated from *Taxus brevifolia*) [5,6]. New natural sources are still being identified, offering perspectives for the development of substances with improved therapeutic outcomes [7]. While several cancer therapies are clinically available, an ideal anticancer drug has not been discovered yet, and numerous side effects of the classical chemotherapeutic drugs limit treatment. Research on new drugs has revealed a variety of new chemical structures with potent biological activities that are of interest in the context of cancer treatment [8,9,10,11]. 

Monoterpenes are the most widespread naturally occurring organic chemicals, being the main constituents of essential oils [12,13,14]. They show antioxidant, antimicrobial, analgesic, anxiolytic, and anticancer properties, and they are of interest as a source of therapeutic alternatives [15,16,17]. Perillyl alcohol, a naturally occurring monoterpene found in the essential oils of peppermint and lavender, has been widely studied [18], demonstrating effectiveness against a variety of human tumor cell lines [19,20,21]. The monoterpene, which previously showed cytotoxic and antitumor activities in various experimental models, is already in clinical trials, mainly for cancer treatment [19,22]. The cytotoxicity of perillyl alcohol analog compounds, namely (−)-8,9-perillaldehyde epoxide, (−)-perillaldehyde, (+)-limonene 1,2-epoxide, and (−)-8-hydroxycarvotanacetone, has also been thoroughly characterized [19].

Among them, limonene has a wide range of applications. Limonene is a potent, naturally occurring antinociceptive and antitumoral compound found in citrus fruits (such as oranges, grapefruit, lemon, lime), as well as in the seeds of caraway and dill [23]. It is also reported to be one of the most abundant terpenes in cannabis, quantified to represent as much as 16% of the essential oil fraction [24]. It is used in the cosmetic industry and perfumery for aromatic purposes, in dermatological applications to induce percutaneous transfer of drugs, and as a sweetener in foodstuffs [25]. Its cytotoxic profile has already been characterized [26], while to exhibit its pharmacological effects, in vitro studies report the need for high concentrations of the terpene.

Solid lipid nanoparticles (SLNs) are composed of lipids that melt above 40 °C to ensure their capacity to modify the release profile in vivo [27]. Besides, due to their composition of biocompatible and biodegradable lipids they have very limited toxicological risk [28,29,30], and can act as penetration (skin) [31] or absorption (oral) [10,32,33] enhancers. Due to their lipid character and solid matrix, SLNs are particularly interesting for topical and oral administration [34]. SLNs are composed of lipids similar to those existing both in the skin sebum and in food, which increases their biocompatibility. When topically applied, SLNs create a thin lipid film onto the surface of the skin, acting as occlusive barrier against water evaporation keeping the skin moist. Hydrated skin is more permeable to active ingredients. Besides, their nanometric size (50–1000 nm) promotes deeper skin penetration and a more homogeneous distribution of the payload. The small-sized particles improve cell uptake and subcellular traffic, and ensure the therapeutic action of essential oil at extracellular and intracellular levels. Topical administration of essential oils is commonly reported for wound healing, hydration, elasticity, scar treatment, and rejuvenation [35]. Their loading into SLNs improves the fixation of the essential oil on the skin, facilitating its penetration. The effects of skin hydration and elasticity were evaluated using the essential oil of rosemary (*Rosmarinus officinalis* L.) encapsulated in solid lipid nanoparticles [36]. Then, the nanoparticles were incorporated into a hydrogel that was applied for one week to the skin of the hands of healthy volunteers, twice a day. The effect of the developed formulation on the hydration of the skin was compared to a hydrogel formulation containing non-encapsulated essential oil. A considerable increase in skin hydration and elasticity was observed with the hydrogel containing essential oil-loaded SLNs. SLNs are also used as absorption enhancers upon oral drug delivery [32,37,38]. SLNs can be proposed to improve the oral bioavailability of essential oils, as the particles undergo metabolic pathways similar as lipids from foods; their reduced size increases passive cellular absorption. 

Considering its anticancer activity, the aim of this study was to develop solid lipid nanoparticles (SLNs) for (+)-limonene 1,2-epoxide (*Lim*-SLNs), and test the effect of the particles on the release profile, antioxidant activity, and cytotoxicity in a keratinocyte cell line. Several reports confirm that (+)-limonene 1,2-epoxide can induce tumor cell apoptosis, as well as suppression of the PI3K/Akt/mTOR17 and NF-κB pathways [39]. Phase I clinical trials have been reported by Vigushin et al. for this monoterpene derivative [40].

## 2. Results and Discussion

Knowing that natural products are important sources of new chemical entities with improved biological properties [41], (+)-limonene 1,2-epoxide has been selected from structurally correlated *p*-menthane derivatives described by Andrade et al. [19] to be loaded into glycerol monostearate SLNs. The solid lipid has been selected from preliminary studies published elsewhere [42]. *Lim*-SLNs had a mean size of 194 ± 3.4 nm (PI 0.244), while blank SLNs (*Lim*-free) were of 203 ± 1.5 nm (PI 0.213). For topical administration, a mean particle size of up to 200 nm is desired to reach the dermis [43]. The polydispersity index is also important because it shows a homogeneous size distribution of particles, and for skin administration a value of below 0.4 is desirable. The encapsulation efficiency (EE%) and loading capacity (LC%) reached 63.12 ± 1.43% and 0.39 ± 0.02%, respectively, attributed to the high volatility of the compound. Similar results with peppermint [44] and Yuxingcao [45] essential oils have been reported.

**Figure 1:**
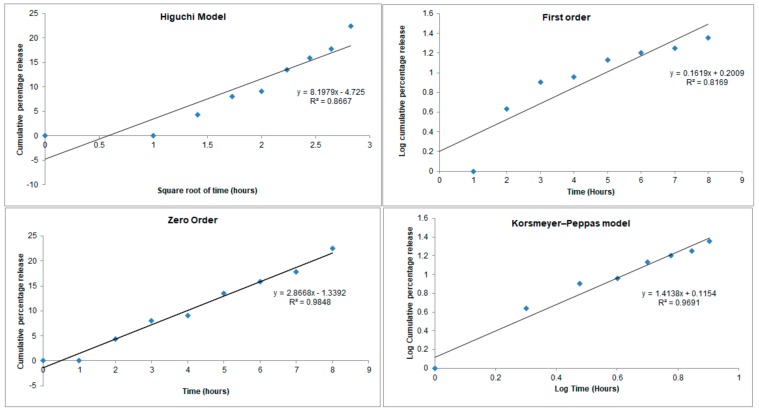
Mathematical fitting models of the release profile of (+)-limonene 1,2-epoxide from solid lipid nanoparticles (SLNs).

The release profile of *Lim*-SLNs was recorded over a period of 8 h, and the obtained values were fitted into four mathematical models (Figure 1).

Less than 5% of limonene was released from SLNs within the first two hours, and after 8 h only 22.5% of the cumulative amount was quantified in the receiving medium of Franz diffusion cells, which translates a delayed release. With respect to the R^2^ values, the best fitting model was shown to be the zero model, with an R^2^ of 0.9848. In such a profile, the drug is released at a constant rate that is only a function of time, typically seen in controlled-release formulations and modeled as: $M_{0}-\;M_{t}=\;k_{0}t$, where $M_{0}$ is the initial concentration of the drug (at time 0), $M_{t}$ is the cumulative amount of drug released at time *t*, and $k_{0}$ is the zero-order release constant with units of concentration per time. The second-best fitting model was the Korsmeyers–Peppas model (Power Law) with a R^2^ of 0.9691, which was developed to specifically describe the release of a drug from a matrix accordingly to $M_{t}/M_{\infty }\;=\;k^{\prime }t^{n}$, where $M_{t}\;$ is the cumulative amount of drug released at time *t*, $M_{\infty }$ is the cumulative amount of drug released at infinite time, $k^{\prime }$ is the constant that is governed by the physicochemical properties of the nanoparticle matrix, and *n* is the diffusional release exponent. In such a model, the *n* value describes the drug release mechanism i.e., if *n* = 0.5 Fickian diffusion is observed, while 0.5 < *n* < 1.0 stands for non-Fickian diffusion. The morphology of the nanoparticles also has a role in the release mechanism. It has been described that in spherical particles as SLNs, drug release becomes independent of time and reaches zero-order release known as Case II transport, achieved as *n* approaches 1.0. This occurs with our *Lim*-SLNs. In such cases, a diffusional exponent *n* = 1.0 is translated non-Fickian transport, whereas super Case II transport is followed in case of *n* > 1.0 [46].

**Figure 2:**
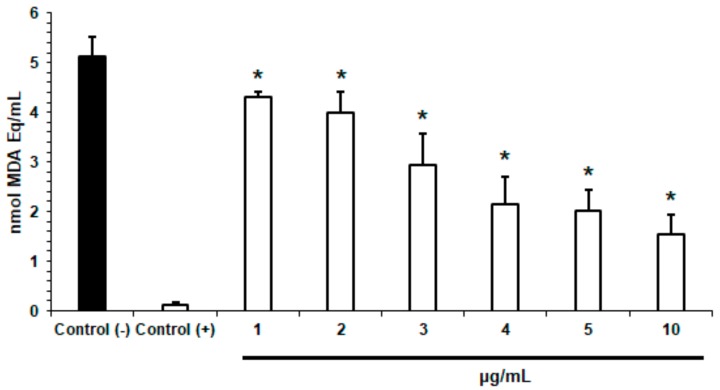
Effect of (+)-limonene 1,2-epoxide and glycerol monostearate solid lipid nanoparticles (*Lim*-SLNs; 1, 2, 3, 4, 5, and 10 µg/mL) on the amount of malondialdehyde equivalents (MDA Eq.) produced in the presence of the free radical FeSO_4_ inducers. Trolox and water were used, respectively, as the positive control and the negative control. Data are presented as mean ± SEM (*n* = 3). The (*) represents statisctical significant values (*p* < 0.05) when compared to the negative control. One-way ANOVA with Dunnet post-test was applied.

The effect of *Lim*-SLNs on the lipid peroxidation was tested at six different concentrations (Figure 2). At the tested concentrations (1, 2, 3, 4, 5, and 10 µg/mL), *Lim*-SLNs inhibited the formation of MDA in a dose-dependent fashion. The obtained MDA values ranged from 4.31 ± 0.11 nmol Eq/mL to 1.55 ± 0.39 nmol Eq/mL (Figure 2), demonstrating the formulation’s capacity to inhibit the Fenton reaction. As lipid peroxides are able to propagate further production of reactive oxygen species (ROS), or degrade producing other reactive compounds capable of crosslinking DNA and proteins, the shown antioxidant activity may also indicate that these particles are able to limit the risk of protein and DNA damage by reactive oxygen species (ROS) [29,47,48].

The antioxidant capacity of *Lim*-SLNs was further tested as a measure to capture free radicals, using the diphenyl-β-picrylhydrazyl (DPPH) test (Table 1). The results show a concentration-dependent behavior. The positive control (butylated hydroxytoluene, BHT), at the highest tested concentration (6.0 µg/mL), showed 77.02% DPPH radical scavenging capacity, and the antioxidant activity was obtained as the correlation between the absorbance decay of the sample test with the absorbance decay of the control test. By plotting the obtained results, a linear regression (y = 4.2026x − 2.644) of *R*^2^ = 0.9902 was obtained and the IC_50_ calculated as 207.5 µg/mL.

**Figure 3:**
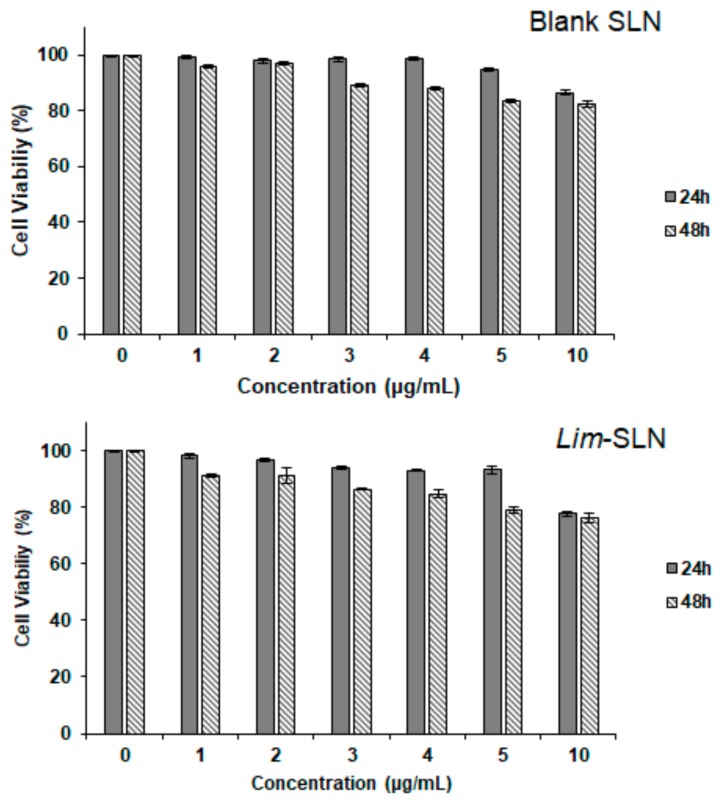
Evaluation of the cytotoxic activity of blank SLNs (top) and of *Lim*-SLNs (bottom), tested at six different concentrations (1, 2, 3, 4, 5, and 10 µg/mL) in the HaCaT cell line using the 3-(4,5-dimethyl-2-thiazolyl)-2,5-diphenyl-2H-tetrazolium bromide (MTT) assay, at 24 and 48 h. Values are mean ± S.D. (*n* = 4).

Previous studies have demonstrated the cytotoxicity and anti-tumor activity of (+)-limonene 1,2-epoxide in different cell lines (OVCAR-8, HCT-116, and SF-295), with percentages of growth inhibition varying between 58.48% (SF-295 cells) and 93.10% (OVCAR-8 cells) [26]. We anticipated that the loading of limonene into SLNs could reduce the cytotoxic effect in cells while keeping the antioxidant activity, as shown in Figure 2. The cytotoxicity assay in the HaCaT cell line showed cell viability above 82.41 ± 0.93% after 48 h for blank SLNs at the highest concentration (10 µg/mL). The loading of limonene into SLNs decreased cell viability down to 76.27 ± 1.63% (10 µg/mL, at 48 h incubation), while remaining above 70%, which is required to confirm the non-cytotoxic profile. Compared with previous studies [26], in which tumoral cell lines were used, in this study (Figure 3) the growth inhibition was below 30%, which may indicate a low toxic effect to the non-tumoral cell line HaCaT. The selective effect of (+)-limonene 1,2-epoxide is of interest in several areas of knowledge, deserving further deeper study. Also, the improved cell resistance was attributed to the modified release profile of the terpene loaded into SLNs and the relatively high encapsulation efficiency within the SLN matrix. Besides, (+)-limonene 1,2-epoxide is highly volatile; its loading into SLNs offers an approach to control its release profile (as demonstrated in Figure 1), reducing its cytotoxic events in the cells, as also shown for other monoterpene derivatives [49,50].

## 3. Materials and Methods

### 3.1. Materials 

(+)-Limonene 1,2-epoxide, a mixture of cis/trans-isomers, ≥97.0% (sum of isomers, GC; PubChem ID 57647614; CAS Number 203719-54-4), was purchased from Sigma Aldrich (Sintra, Portugal). Imwitor^®^900K was received as a gift from Cremer Oleo GmbH & Co. KG company (Hamburg, Germany). Poloxamer^®^ 188 (a non-ionic triblock copolymer composed of central hydrophobic polyoxypropylene chain (PPOx, where x = 28) and by two hydrophilic chains of polyoxyethylene (PEOy, where y = 79)), was purchased from BASF (Ludwigshafen, Germany). Phosphate-buffered saline (PBS; pH 7.40) was from Sigma-Aldrich (Sintra, Portugal). The other chemicals, i.e., 3-(4,5-dimethyl-2-thiazolyl)-2,5-diphenyl-2H-tetrazolium bromide (MTT), doxorubicin (purity > 98%), Trolox, thiobarbituric acid (TBA), butylated hydroxytoluene (BHT), and dimethyl sulfoxide (DMSO), were purchased from Sigma Chemical Co. (St. Louis, MO, USA). Double-distilled water (MilliQ) was used after filtration in a Milli-Q^®^ Plus system (Millipore, Germany).

### 3.2. Production of SLNs and Lim-SLNs

The production of SLNs was carried out by hot high-pressure homogenisation [42], using Imwitor^®^900K (glycerol monostearate) as solid lipid and Poloxamer^®^ 188 as surfactant. Briefly, the melted lipid phase consisting of Imwitor^®^900K (5% (*w*/*v*)) was dispersed in an aqueous phase consisting of Poloxamer^®^ 188 (2.5% (*w*/*v*)) and MilliQ water, at 70 °C. A pre-emulsion was produced by stirring the mixture at 8000 rpm for 30 s in an Ultra-Turrax (Ultra-Turrax^®^, T25, IKA). The obtained pre-emulsion was poured into the high-pressure homogenizer (EmulsiFlex^®^-C3, Avestin), previously heated by recirculating hot Milli-Q water at 75 ± 0.5 °C, for 5 min, applying 500 bars in the first cycle and 60 bars in the second cycle. Then, after 5 min in the homogenizer, the obtained *o*/*w* nanoemulsion was transferred to siliconized glass vials and was allowed to cool down in the fridge (4 ± 0.5 °C) to generate SLNs. Imwitor^®^900K (4.5% (*w*/*v*)) and (+)-limonene 1,2-epoxide (0.5% (*w*/*v*)) were used to produce *Lim*-SLNs as described, by adding the drug to the melted solid lipid before the production of the pre-emulsion.

### 3.3. Mean Particle Size and Polydispersity Index

Dynamic light scattering (DLS, Zetasizer Nano ZS, Malvern, Worcestershire, UK)) was used to determine, immediately after production, the mean particle size (z-Ave) and polydispersity index (PI) of SLNs and *Lim*-SLNs. Prior to analysis, aqueous nanoparticle dispersions were diluted 100-times in MilliQ water, and analyzed in triplicate measurements (*n* = 3) (10 runs per measurement, 30 in total). Data are expressed as the arithmetical mean ± standard deviation (SD).

### 3.4. Encapsulation Efficiency (EE) and Loading Capacity (LC)

The encapsulation efficiency (EE) and loading capacity (LC) of (+)-limonene 1,2-epoxide in SLNs were calculated as follows [33]:

(1) $$EE\%=\;\frac{W_{Lim}-W_{s}}{W_{Lim}}\;\times 100$$

(2) $$LC\%=\;\frac{W_{Lim}-W_{s}}{W_{Lim}-W_{s}+W_{L}}\;\times \;100$$

where *W_Lim_* is the mass of (+)-limonene 1,2-epoxide used for the production of SLN, *W_L_* is the mass of lipid added for the production of SLNs, and *W_Lim_* is the mass of (+)-limonene 1,2-epoxide quantified in the supernatant. Briefly, *Lim*-SLNs were firstly ultra-centrifuged for 1 h at 100,000× *g* in a Beckman Optima™ Ultracentrifuge (Optima™ XL, Indianapolis, IN, USA) and with quantification determined in the supernatant in a UV spectrophotometer Shimadzu UV-1601 (Shimadzu Italy, Cornaredo, Italy) at 290 nm.

### 3.5. In Vitro Release Profile of Lim-SLN

The in vitro release profile of (+)-limonene 1,2-epoxide from SLNs (*Lim*-SLNs) was determined using vertical Franz glass diffusion cells. A cellulose membrane from MERCK KgaA (Darmstadt, Germany), with an average pore size of 0.22 µm, was firstly soaked for 2 h in phosphate-buffered saline (PBS, pH 7.4), and then placed in between the donor and acceptor chambers (*n* = 3/sample). A volume of 1 mL of freshly prepared *Lim*-SLNs was placed onto the hydrated cellulose membrane. The acceptor chamber, containing 5 mL of PBS buffer, was kept under magnetic stirring at 37 °C over the course of the assay. At pre-determined time-intervals, a volume of 200 µL was sampled with a syringe, with the same volume replaced with PBS buffer to maintain sink conditions. The amount of released (+)-limonene 1,2-epoxide was analysed in a UV spectrophotometer Shimadzu UV-1601 (Shimadzu Italy, Cornaredo, Italy), at 290 nm for the quantification of limonene. For the mathematical fitting, four kinetic models (zero-order, and first-order kinetics, Higuchi and Korsmeyer–Peppas), have been used [51]. The selection of the most appropriate model was based on the obtained R^2^ values.

### 3.6. In Vitro Lipid Peroxidation Assay

Increasing concentrations of *Lim*-SLNs (1, 2, 3, 4, 5, and 10 µg/mL) were added to a mixture of 1 mL of egg yolk homogenate (1% *w*/*v*) in phosphate buffer (pH 7.4) and 0.1 mL ferrous sulphate (FeSO_4_, 0.17 mol/L). The obtained mixture was incubated for 30 min at 37 °C. Upon cooling, a volume of 0.5 mL was centrifuged with 0.5 mL of trichloroacetic acid solution (15 wt%) at 1200 rpm for 10 min. The collected supernatant (0.5 mL) was mixed with the same volume of thiobarbituric acid solution (0.67 wt%) and incubated for 60 min at 95 °C. After cooling, the formation of thiobarbituric acid reactive substance (TBARS) was quantified by spectrophotometry by measuring the supernatant absorbance at 532 nm and the results are expressed as malondialdehyde equivalents (MDA Eq) of the substrate. Trolox (standard antioxidant) at 50 μg/mL was set as the positive control and water as the negative control.

### 3.7. In Vitro Antioxidant Activity Against Free Radical DPPH

The capacity of the antioxidants present in *Lim*-SLNs to scavenge the stable radical DPPH^•^ was translated as the antioxidant activity of the sample test [52]. Briefly, several solutions were prepared by dissolving *Lim*-SLNs in a methanolic solution of 0.1 mM DPPH to achieve concentrations of solid lipid of 1, 2, 3, 4, 5, and 10 µg/mL. A volume of 20 µL of each prepared sample was placed in the microplate wells. Finally, a volume of 200 µL DPPH methanolic solution (0.1 mM) was added to each well. Methanol was used as the negative control and butylated hydroxytoluene (BHT, 0–6 µg/mL) as the positive control. The microplates were incubated at 25 °C for 30 min, and then read in a multiplate reader (DTX 880 Multimode Detector, Beckman Coulter Inc.) at 517 nm. The antioxidant activity or the percentage of scavenging of free radicals was calculated from the optical density (OD) of the negative control (methanol) in comparison to the sample test (*Lim*-SLNs) using the following Equation:

(3) $$Antioxidant\;activity\;\left(\%\right)=\frac{OD_{Methanol}\;-OD_{Lim-\mathrm{SLN}}}{OD_{Methanol}}\;\times 100$$

By the end of the assay, the IC_50_ values were calculated through linear regression (r^2^ = 0.965) by plotting the percentage of scavenging in the Y-axis (% inhibition) against the concentration in the X-axis (μg/mL).

### 3.8. Cell Culture and MTT Assay

The cytotoxicity of SLNs (blank) and *Lim*-SLNs was tested in HaCaT cells, purchased from ATCC (LGC Standards S.L.U., Barcelona, Spain [53]). Cells were cultured in RPMI-1640 medium supplemented with 10% fetal bovine serum, 2 mM L-glutamine, 100 µg/mL streptomycin, and 100 U/mL penicillin, and further incubated at 37 °C in a 5% CO_2_ atmosphere. Consumables for cell culture were obtained from Sigma Chemical Co. (St. Louis, MO, USA). For the 3-(4,5-dimethyl-2-thiazolyl)-2,5-diphenyl-2H-tetrazolium bromide (MTT) assay [54], cells were incubated in 96-well plates (0.1 × 10^6^ cells/mL, 100 μL/well in culture medium) for 24 h. Solutions of SLNs (blank) and *Lim*-SLNs in dimethyl sulfoxide (DMSO 0.7%) at increasing concentrations (1, 2, 5, and 10 μg/mL) were prepared in fetal bovine serum (FBS)-free culture medium, added to each well, and then incubated for more 72 h at 37 °C in a 5% CO_2_ atmosphere. A solution of DMSO 1% was set as the negative control and doxorubicin at 100 μg/mL as the positive control (doxorubicin: purity > 98%; Sigma Chemical Co., St. Louis, MO, USA). At the end of incubation time the test solution was removed and replaced by 150 μL of MTT (0.5 mg/mL), added to each well, and incubated for 3 h at 37 °C in a 5% CO_2_ atmosphere. Cell viability was assessed by the ability of viable cells to reduce the yellow dye MTT to the purple formazan. The obtained precipitate was then dissolved by adding 150 μL DMSO to each well, and the absorbance was read at 595 nm using a multiplate reader (DTX 880 Multimode Detector, Beckman Coulter Inc.). The results were expressed as percentage of cell growth inhibition (%GI) as follows:

(4) $$\%GI=100\;\times \;\left[\frac{Abs_{Test}}{Abs_{Negative\;Control}}\times 100\right]$$

### 3.9. Statistical Analysis

The statistical significance of differences between mean values was determined by ANOVA with Dunnet post-test. Results were considered significantly different if *p* < 0.05 (marked with an asterisk (*), if applicable). Graphs and the statistical evaluation were developed using the GraphPad Prism software (Intuitive Software for Science, San Diego, CA, USA). The results are presented as the mean ± standard error of mean (SEM).

## 4. Conclusions

*Lim*-SLNs, composed of glycerol monostearate and Poloxamer^®^ 188, have been successfully produced by high pressure homogenization, with a small size (<200 nm), low polydispersity index (<0.25), and loading capacity and encapsulation efficiency of 0.39% and 63%, respectively,. The release profile of monoterpene from SLNs fitted to the zero-order kinetic model, typical of controlled release formulations. The loading of the monoterpene retained its antioxidant profile. Our findings strengthen the added value of using naturally occurring anti-oxidant and anti-tumoral compounds in innovative formulations, which can be further exploited for several administration routes including the oral and topical routes. Lipid nanoparticles are particularly interesting as drug carriers for skin and oral administration due to their role as penetration and absorption enhancers, as their composition resembles both lipids from human body and from food.

## Figures and Tables

**Table 1:** Evaluation of the percentage of scavenging of free radical DPPH of *Lim*-SLNs tested at six different concentrations (1, 2, 3, 4, 5, and 10 µg/mL).

*Lim*-SLNs (µg/mL)	% Scavenging
1	1.98
2	6.03
3	9.48
4	13.82
5	17.33
10	23.75
